# Frequency of Dyslipidemia in patients with Lupus Nephritis

**DOI:** 10.12669/pjms.332.12410

**Published:** 2017

**Authors:** Saba Sajjad, Sumaira Farman, Muhammad Ahmed Saeed, Nighat Mir Ahmad, Bilal Azeem Butt

**Affiliations:** 1 Saba Sajjad, FCPS (Medicine), Division of Rheumatology, Fatima Memorial College of Medicine & Dentistry, University of Health Sciences, Lahore, Pakistan; 2 Sumaira Farman, FRCP, FACP, FACR, SCE Rheumatology, Graduate Certificate Pediatric Rheumatology, Division of Rheumatology, Fatima Memorial College of Medicine & Dentistry, University of Health Sciences, Lahore, Pakistan; 3 Muhammad Ahmed Saeed, FCPS (Rheumatology) FACR, FCPS (Medicine), Division of Rheumatology, Fatima Memorial College of Medicine & Dentistry, University of Health Sciences, Lahore, Pakistan; 4 Nighat Mir Ahmad, FACP, FACR, DABR, DABIM, Division of Rheumatology, Fatima Memorial College of Medicine & Dentistry, University of Health Sciences, Lahore, Pakistan; 5 Bilal Azeem Butt, FCPS (Medicine), Division of Rheumatology, Fatima Memorial College of Medicine & Dentistry, University of Health Sciences, Lahore, Pakistan

**Keywords:** Dyslipidemia, Lupus Nephritis, Systemic Lupus Erythematosus (SLE)

## Abstract

**Objective:**

To determine the frequency of dyslipidemia in patients with lupus nephritis and its association with the degree of proteinuria.

**Methods:**

This cross-sectional analytic study included 65 patients who fulfilled the ACR (American College of Rheumatology) criteria for SLE and had renal involvement, presenting to the Division of Rheumatology, Fatima Memorial Hospital (FMH), and Lahore from 21^st^ Sep 2016 to 20^th^ Dec 2016. After 12 hours overnight fast their blood samples were assessed for total cholesterol (TC), triglycerides (TG), high density lipoprotein (HDL) and low density lipoprotein (LDL). Patient demographic variables (age, sex) and disease characteristics (disease duration, degree of proteinuria, steroid dose) were noted. Patients were categorized into two groups on the basis of degree of proteinuria: having proteinuria >1gm or ≤ 1gm. Data was analyzed using SPSS version 22. Individual lipid profiles were correlated with the degree of proteinuria.

**Results:**

Most common lipid abnormality found in our study was hypertriglyceridemia (58.5%). Total Cholesterol and LDL-C was high in 55.4% and 30.8% subjects respectively. Low HDL was found in 21.5% subjects. Increased frequency of dyslipidemia was noticed in those subjects who had proteinuria >1gm (P value < 0.05).

**Conclusion:**

Dyslipidemia was observed in a high frequency in patients with lupus nephritis and was strongly associated with their degree of proteinuria.

## INTRODUCTION

Systemic lupus erythematosus (SLE) is the prototypic autoimmune disease characterized by multisystem involvement and the production of an array of autoantibodies.^1^ SLE may involve any organ mainly skin, joints, kidneys, heart, lungs and central nervous system.^2^ Most of the patients are young females with F/M ratio of 15.5:1.^3^ Presenting features may vary from skin and joint involvement to organ and life-threatening complications like lupus nephritis (33%).^3^

Lupus is associated with pre-mature atherosclerosis owing to lipid abnormalities which are common in these patients. Most studies reveal that relative risk of myocardial infarction exceeds 5 to 8 times compared to general population.^4^ Studies have shown that the prevalence of dyslipidemia in lupus patients ranges from 36% at diagnosis to 60% or even higher after 3 years.^5^ Patients with SLE have an elevated plasma TG, LDL- C, Apoprotein B, and decreased HDL-C.^6^ Elevated TG levels in SLE patients are in part attributable to anti-lipoprotein lipase (anti-LPL), which are present in 47% of patients.^7^

Dyslipidemia is believed to decisively affect the long-term prognosis of lupus patients, not only with regard to cardiovascular events but also by influencing lupus nephritis.^5^ Nephrotic-range proteinuria, elevated TC level and decreased serum albumin levels not only reflect the activity but also the severity of renal damage in SLE patients.^8^ Studies have also shown higher degree of dyslipidemia prevalence in patients having a disease duration of 3 years, Max-SLEDAI ≥ 2 and taking Prednisone ≥ 30 mg/d.^9^

Hyperlipidemia and lipoprotein abnormalities may play a role in development of glomerular atherosclerosis in renal disease.^10^ Dyslipidemia is prevalent and more severe in lupus nephritis (LN) patients as compared to controls with a similar degree of chronic kidney disease (CKD) despite disease quiescence, low steroid dose and low level of proteinuria.^11^ It has been seen that concomitant corticosteroids and renal dysfunction increases the severity of dyslipidemia while Hydroxychloroquine (HCQ) reduces the risk.^11^ In patients with LN, hypertension, hyperlipidemia and antiphospholipid syndrome are important risk factors associated with a higher mortality rate and development of renal failure.^12^ Statin therapy in lupus patients reduces the risk of mortality (HR 0.44, 95% CI 0.32 to 0.60); coronary artery disease (CAD) (HR 0.20, 95% CI 0.13 to 0.31); cerebrovascular disease (CVD) (HR 0.14, 95% CI 0.08 to 0.25) and end-stage renal disease (ESRD) (HR 0.22, 95% CI 0.16 to 0.29).^13^

Dyslipidemia in lupus patients is often under-recognized and also under-treated. Lipid abnormalities lead to pre-mature atherosclerosis which in turn leads to pre-mature CAD in lupus patients. Persistent dyslipidemia is also an independent risk factor to predict the development of CKD in LN patients. Therefore, lipid profile should be monitored regularly and dyslipidemia should be managed aggressively to prevent deterioration of kidney function in such patients. This study was undertaken as there is no data on the prevalence of dyslipidemia and its association with proteinuria in lupus nephritis patients from Pakistan.

## METHODS

The study was conducted in the Division of Rheumatology, FMH Lahore, from September to December 2016, after approval from Institutional Review Board (IRB), FMH, and Lahore. A total of 65 patients were selected both from outpatient and inpatient departments after calculating the sample size (95 % Confidence level, 10 % margin of error and taking frequency of dyslipidemia in SLE of 60%).^7^ All patients were >18yrs old and fulfilled the ACR classification criteria for SLE with renal involvement either biopsy proven or had proteinuria, hematuria or an elevated serum creatinine. Patients with CKD, diabetes mellitus, CAD, essential hypertension, Liver or thyroid disease or having h/o familial hyperlipidemia were excluded. Patients on lipid lowering therapy were also not included. Written informed consent was taken from each patient for participation in the study and confidentiality was maintained. Their demographic profiles (i.e. age, sex), disease characteristics (disease duration, degree of proteinuria, current dose of steroids and HCQ) and renal biopsy findings were also noted using a structured questionnaire.

After a 12 hours overnight fast and consumption of normal diet for previous two weeks (without fat restriction) blood samples were assessed for TC, TG, HDL and LDL-C. Hyperlipidemia was diagnosed according to National Cholesterol Education Program (TC >200mg/dl, TG >150mg/dl, LDL-C >130mg/dl, HDL-C <40mg/dl).^14^

The data was analyzed using SPSS version 22.0. Age, BMI, disease duration, P:C ratio, steroid dose, HCQ dose, TC, TG’s, LDL and HDL were presented as mean and ± standard deviation. Categorical variables like gender, degree of proteinuria and dyslipidemia were presented as percentage. On the basis of amount of proteinuria patients were divided into two groups: having a spot urinary protein/creatinine ratio (P: C ratio) of > 1.0 or P:C ratio ≤ 1.0. These two groups were compared for their degree of dyslipidemia by using chi-square test. Individual parameters of fasting lipid profiles were correlated with the degree of proteinuria by Pearson correlation curve. Steroid dose, HCQ dose, BMI and disease duration were also compared with individual lipid profiles using chi-square test. P value ≤ 0.05 was considered significant.

## RESULTS

Total patients were 65 with 83.1% (n=54) females. Demographic details and disease characteristics are shown in Table-I. In our study 55.4% (n=36) of the subjects had TC > 200mg/dl, 58.5% (n=38) had TG’s >150mg/dl, and 30.8% (n=20) had LDL > 130mg/dl. 21.5% (n=14) of subjects had HDL levels <40mg/dl. Proteinuria >1gm was present in 55.4% (n=36), while 44.6% (n=29) had proteinuria <1gm.

**Table-I:** Demographic details and disease characteristics.

*Parameter*	*Mean ± SD*
Age (years)	27.00± 8.00
Body mass index (kg/m^2^)	22.88± 4.47
Duration of symptoms (years)	3.02± 1.97
Creatinine (mg/dl)	1.69± 2.00
Albumin (mg/dl)	3.35± 0.45
Spot urine protein spot urine creatinine ratio	1.55± 0.50
Cholesterol (mg/dl)	210.26± 42.47
Triglycerides (mg/dl)	204.77± 90.54
HDL-C (mg/dl)	47.91± 8.80
LDL-C (mg/dl)	112.38± 25.71
Steroid dose (mg/day)	11.36± 11.26
HCQ dose (mg/day)	276.92± 93.16

**Fig.1:**
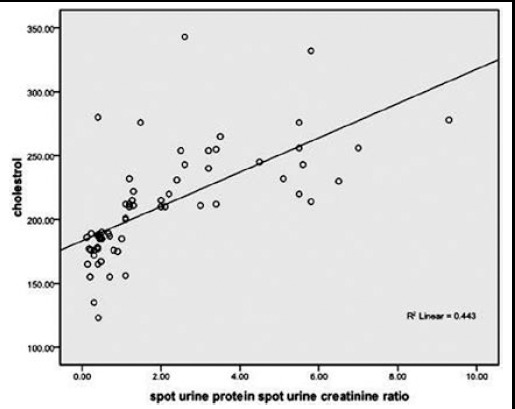
Correlation of cholesterol and Proteinuria.

**Fig.2:**
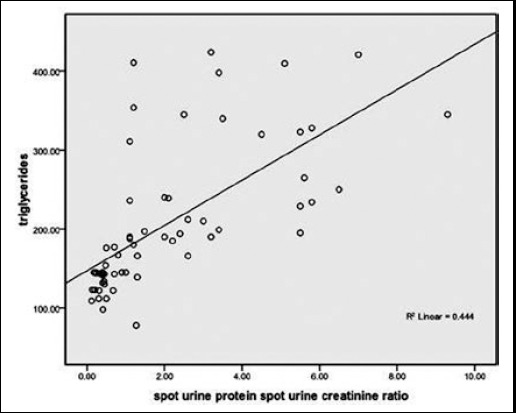
Correlation of triglycerides and Proteinuria.

**Fig.3:**
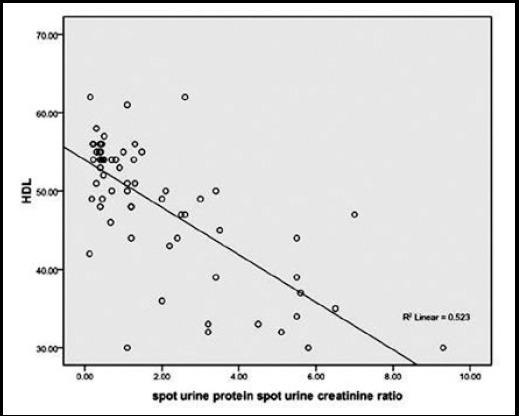
Correlation of HDL and Proteinuria.

**Fig.4:**
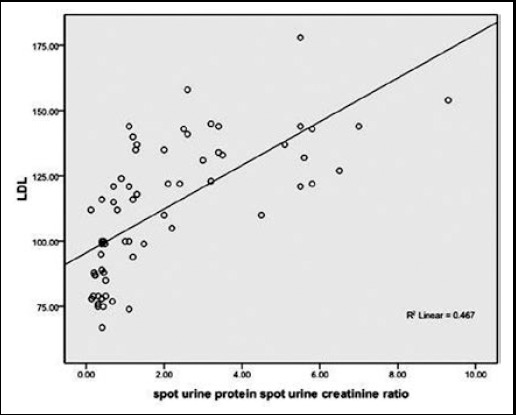
Correlation of LDL and Proteinuria.

Frequency of patients with high TC, TG’s, LDL was found to be significantly lower in sub-groups of patients with proteinuria ≤ 1gm and none of the patients in this sub-group had HDL <40mg/dl.(Table-II). Significant positive correlation was found between TC, TG’s, LDL and proteinuria respectively, whereas significant negative correlation was found between HDL and proteinuria as shown in Fig.1-4. TC was also found to be positively correlated with the BMI (Chi-square=4.142 P < 0.042), disease duration >3yrs (Chi-square=16.984 P < 0.05) and steroid dose >10mg/day (Chi-square=13.97 P < 0.001) while negatively correlated with HCQ dose > 200mg/d (Chi-square=6.987 P <0.00

**Table-II:** Relationship of lipid profile to degree of proteinuria.

	*P:C ratio >1.0 n (%)*	*P:C ratio ≤1.0 n (%)*	*P-value*
TC>200mg/dl	35 (97.2%)	1 (2.8%)	<0.001
TG’s>150mg/dl	34 (89.5%)	4 (10.5%)	<0.001
HDL<40mg/dl	14 (100%)	0 (0.0%)	<0.001
LDL>130mg/dl	20 (100%)	0 (0.0%)	<0.001

## DISCUSSION

Premature coronary artery disease (CAD) significantly affects morbidity and mortality in SLE. Certain traditional and disease-specific factors have been identified as independent predictors for CAD. Among the former are age (particularly postmenopausal state), male sex, arterial hypertension, dyslipidemia, and smoking. Disease activity and duration, cumulative damage, antiphospholipid antibodies, high sensitivity C-reactive protein, and renal disease are the most consistent disease-related factors. Corticosteroids are linked to increased CAD risk whereas antimalarial are protective.^15^

In our study the mean age was 27 with majority (83%) being females. All patients had renal involvement either biopsy proven (78%) or manifested urinary abnormalities during the course of their illness. At the time of study proteinuria was present in 86% (nephrotic range in 26%), microscopic hematuria in 56.8%. Hypertension was present in 60%. Some of the results are similar to those reflected in previous local studies which reported lupus nephritis being commoner in females (92%); commonest age is 20-40years. In these studies proteinuria had been reported in 74% (nephrotic range in 45%), microscopic hematuria in 66.6% and hypertension was found to be present in 70%.^16^-^18^ In our study Mean TC was 210.3±42.5, TG 204.8±90.5, HDL 47.91±8.8, LDL 112.4±25.7. Kakati S et al. reported that out of 30 SLE patients 19 had dyslipidemia as compared to 5 of the controls with Mean cholesterol of 155.8±43.4, TG 222.6±49.9, LDL 132.8±74.2, HDL 37.4±3.8.^19^

In our study the proportion of high TC, TG’s and LDL was 55.4%, 58.5% & 30.8% respectively and low HDL 21.5%. Wijaya LK et al. reported that 75.3% of lupus patients have dyslipidemia, with high TC, TG’s, LDL in 43%, 44.2%, 26.4% respectively and low HDL-C in 26%.^9^ Radillo HA et al. found out that 68.8% of lupus patients had dyslipidemia which was associated with disease activity measured by SLEDAI, presence of lupus nephritis, use of prednisolone >20mg/d, evolution of disease <3years, while absence of dyslipidemia with the use of HCQ.^20^ We have also found a positive correlation between dyslipidemia and prednisolone dose>10mg/d (mean steroid dose was 11.36+11.26), disease duration>3yrs while low TC when on adequate doses of HCQ. A Chinese study reported that 59% of LN patients and 46% CKD controls showed dyslipidemia. LN patients showed higher TC and TG’s than controls. More LN patients had abnormal TC, TG or LDL-C (54%, 16% and 38% respectively).^10^

We in our study noticed a positive correlation between dyslipidemia and proteinuria. HN Reich et al. found a statistically important interaction between cholesterol and proteinuria (p=0.009).^21^ Similarly Kyung-Eun Lee et al. studied 68 patients with biopsy proven lupus nephritis. He found out that the group having higher LDL-C >=100mg/dl excreted more 24hour urine protein than lower LDL-C group. The higher LDL-C was a significant predictor of CKD in these patients on follow-up.^22^ Lui L et al. reported that SLE patients had significantly higher TC, TG, LDL-C levels and significantly lower HDL-C levels compared with the control group (all P<0.01). TC, TG and LDL-C levels were positively correlated with lupus nephritis, corticosteroids therapy and 24 hours urine protein content, HDL-C levels were positively correlated with age, lupus nephritis, and corticosteroids therapy.^23^

In lupus nephritis both cholesterol and proteinuria have been repeatedly shown to be interrelated and modifiable risk factors for pre-mature CAD and progressive loss of kidney function. Traditionally care has always been emphasized to control the disease flares, and less attention has been paid to screening and treating patients for dyslipidemia. So it is imperative for all clinicians treating SLE patients to screen for dyslipidemia and start treatment as early as possible to achieve better disease outcomes and improve quality of life of SLE patients. However our study highlights that apart from instituting lipid lowering therapy, targeting the disease to remission might alone be helpful in modifying this CV risk factor and improving the outcome of lupus patients.

### Limitations of the study

Firstly, there was no control group. Secondly, this was a cross-sectional study. In future we need to plan a long-term prospective study with a larger sample size and a control group to look for association of dyslipidemia with disease activity and disease outcome.

## CONCLUSION

This study has shown that almost half of the patients with lupus nephritis had high total Cholesterol and Triglycerides and one in every third patient had high LDL levels. All these parameters have been found to be significantly associated with the degree of proteinuria.

### Author’s Contribution

**SS, SF & MAS:** conceived the manuscript.

**SS & BAB:** manuscript writing & editing.

**SS & MAS:** data collection and statistical analysis

**SF, MAS & NMA:** reviewed and did final approval of manuscript.
